# Condition-dependent disruption of low-frequency EEG–fMRI coupling reveals delayed hemodynamic timing and motor-network reorganization in chronic stroke

**DOI:** 10.1016/j.nicl.2026.103989

**Published:** 2026-03-28

**Authors:** Parikshat Sirpal, Nishaal Parmar, Beni Mulyana, Hazem H. Refai, Yuan Yang

**Affiliations:** a Department of Neurosurgery, University of Oklahoma Health Sciences Center, Oklahoma City, OK 73019 United States; b Center for Geroscience and Healthy Brain Aging, University of Oklahoma Health Sciences Center, Oklahoma City, OK 73019, United States; c School of Electrical and Computer Engineering, University of Oklahoma, Gallogly College of Engineering, Norman, OK 73019 United States; d Department of Electrical and Computer Engineering, College of Engineering, University of Tulsa, Tulsa, OK 74133 United States; e University of Illinois Urbana-Champaign, Department of Bioengineering, Urbana, IL 61801 United States; f Carle Foundation Hospital, Urbana, IL 61801 United States; g University of Illinois Urbana-Champaign, Beckman Institute for Advanced Science and Technology, Urbana, IL 61820 United States; h University of Illinois Urbana-Champaign, Carle Illinois College of Medicine, Department of Biomedical and Translational Sciences, Urbana, IL 61801 United States

**Keywords:** Neurovascular Coupling, EEG-fMRI, Low-frequency oscillations, Stroke Motor Impairment, Hemispheric Reorganization

## Abstract

Neurovascular coupling (NVC) is frequently disrupted after stroke, yet its temporal alignment and motor-network organization during sensorimotor engagement remain incompletely characterized. We tested whether chronic hemiparetic stroke alters the temporal alignment between EEG low-frequency oscillations (LFOs; 0.1–1.5 Hz) and the fMRI BOLD response during peripheral finger stimulation, and whether these alterations map onto motor-network anatomy and motor impairment. Simultaneous EEG–fMRI was acquired in 13 participants (7 chronic stroke; 6 healthy controls), during block-design peripheral transcutaneous index finger stimulation. EEG was decomposed using empirical mode decomposition to isolate physiologically grounded LFO components and Hilbert amplitude envelopes were incorporated into voxel-wise EEG-informed fMRI models. To evaluate robustness to hemodynamic assumptions, we implemented canonical HRF modeling, a derivative-augmented basis set, and subject-specific HRF estimation. Spatial inference was anchored to the Human Motor Area Template (HMAT). Stroke participants exhibited delayed and attenuated stimulation-evoked BOLD responses relative to controls. Cross-modal timing analyses revealed a distributional shift of peak EEG–BOLD alignment lags in stroke toward smaller and more frequently negative values, consistent with altered temporal alignment in the context of delayed hemodynamics, as opposed to causal inversion. The relative ordering of timing and coupling effects was preserved across HRF modeling strategies. HMAT-constrained analyses demonstrated reduced ipsilesional M1/PMd coupling during paretic stimulation with increased contralesional premotor and SMA recruitment. Importantly, ipsilesional motor-region coupling scaled with upper-extremity Fugl–Meyer scores. Together, these findings demonstrate convergent temporal and spatial reorganization of EEG–BOLD coupling after stroke and support LFO-informed EEG–fMRI as a physiologically interpretable framework for quantifying motor-network neurovascular timing.

## Highlights

• Stroke delays and attenuates event-locked BOLD responses to motor stimulation.
• EEG–fMRI reveals altered temporal alignment of neural oscillations and BOLD in stroke.
• Lag polarity shifts may reflect delayed hemodynamics, but not causal reversal.
• Hemispheric analysis reveals ipsilesional suppression and network reweighting in stroke.
• EEG–fMRI coupling strength tracks motor impairment severity in stroke.

## Graphical abstract

**Figure:**
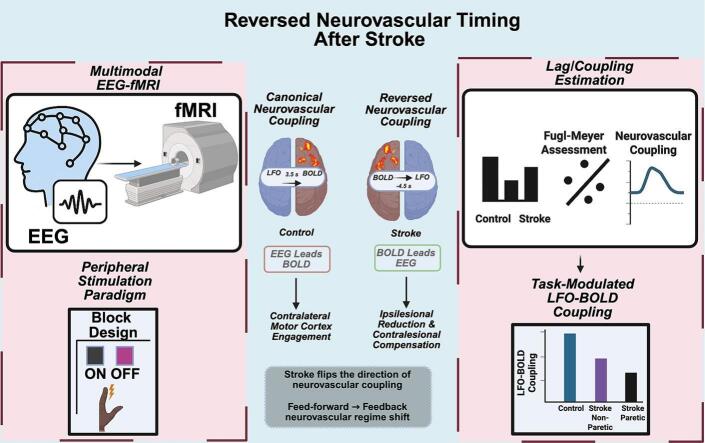

## 1 Introduction

Stroke remains one of the leading causes of adult disability worldwide, with motor impairment representing a primary contributor to long-term functional deficits (Boehme et al., Feb. 2017, Lindsay, 2019”, 2019,) . Despite advances in neurorehabilitation, the biological mechanisms that govern recovery trajectories remain incompletely understood, particularly, the link between spontaneous neural activity, hemodynamic signaling, and sensorimotor stimulation (Reinkensmeyer, Dec. 2016, A. Salek-Haddadi, K. J. Friston, L. Lemieux, and D. R. Fish, “Studying spontaneous EEG activity with fMRI,”, 2003, Fox and Raichle, 2007, Obrig, 2000) . Recent work has emphasized the role of low-frequency oscillations (LFOs), i.e., rhythmic neural activity below 2 Hz, as an important physiological marker bridging neuronal and vascular domains (Obrig, 2000, Laufs, 2003) , as LFOs are observable in both electroencephalography (EEG) and functional magnetic resonance imaging (fMRI), and may serve as a proxy for neurovascular coupling (NVC) (Song, 2016, Nishijima et al., 2016, Zaldivar, 2017, Phillips et al., Nov. 2015, Sirpal et al., 2021) . However, while gross hemodynamic deficits are well-documented, whether the temporal fidelity of neurovascular coupling is maintained across functionally distinct motor subregions remains unknown. This gap in knowledge masks a potential latent vascular instability that may impede sensorimotor recovery even when structural perfusion appears restored.

In the healthy brain, NVC operates as a feedforward mechanism, whereby neuronal depolarization triggers metabolic and astrocytic signaling cascades that result in localized vasodilation and increased cerebral blood flow. This cascade manifests as a canonical hemodynamic delay, with EEG activity typically preceding the peak blood oxygen level dependent (BOLD) signal by approximately 3 to 6 s (Logothetis and Wandell, 2004, Kim et al., 2004) . However, this temporal and spatial fidelity is frequently disrupted in stroke, since lesions affecting cortical gray matter, white matter tracts, or vascular structures may impair NVC integrity, leading to aberrant timing relationships between neuronal and hemodynamic signals. These disruptions may reflect hypoperfusion, endothelial dysfunction, impaired neuro-glial signaling, or the maladaptive reorganization of cortical networks (Wang et al., Jun. 2016, Frizzell et al., 2020, Schulz, Jan. 2015) . However, stroke-related changes are rarely symmetric, as afferent drive from the paretic versus non-paretic limb engages different hemispheric circuits, the integrity of NVC may depend on which motor pathway is recruited (McPherson and Dewald, Oct. 2022). Despite extensive work on hemodynamic response function amplitude and latency in stroke, there is currently no quantitative, region-resolved metric of cross-modal EEG–fMRI timing within the sensorimotor network during behaviorally relevant input. Prior studies have largely relied on BOLD-only measures or global lag estimates, which do not capture spatially heterogeneous neurovascular timing across motor subregions. As a result, it remains unclear whether stroke produces a uniform delay in neurovascular coupling or a region-specific disruption of cross-modal temporal alignment. We frame neurovascular coupling not only in terms of response magnitude but in terms of neurovascular temporal fidelity, defined as the stability of phase alignment between neuronal low-frequency oscillatory envelopes and the corresponding BOLD response. In the healthy brain, this phase relationship is temporally consistent across trials and regions, whereas stroke is hypothesized to produce spatially heterogeneous and temporally unstable coupling.

Peripheral stimulation of the paretic hand predominantly probes the lesioned hemisphere, where neurovascular signaling is expected to be attenuated and temporally disrupted. In contrast, peripheral stimulation of the non-paretic hand engages the relatively preserved contralesional hemisphere, offering a window into spared neurovascular function (Mang et al., Oct. 2023). Separating these conditions allows us to determine if this leads to global NVC changes, or whether intact pathways retain more control-like coupling dynamics. We further distinguish between ipsilesional vascular–neuroglial impairment, which is expected to degrade neurovascular temporal fidelity, and contralesional compensatory recruitment, which may exhibit preserved or even temporally shifted coupling within premotor and supplementary motor regions. This framework predicts distinct timing regimes across hemispheres rather than a uniform delay. Here, we quantify and localize region-specific patterns of cross-modal timing, variability, and phase alignment between EEG-derived LFO amplitude envelopes and BOLD signals in chronic stroke participants and healthy controls during peripheral transcutaneous finger stimulation. By modulating cortical excitability in a controlled manner, finger stimulation can offer a mechanistic explanation of how afferent input affects both electrophysiological and vascular dynamics, particularly within primary and secondary sensorimotor regions, anatomically delineated using the Human Motor Area Template (HMAT) (Lindauer, 2010, Strens et al., Aug. 2004, Mayka et al., Jul. 2006) HMAT provides a validated parcellation of six cortical motor regions (M1, S1, PMd, PMv, SMA, pre-SMA), enabling voxelwise results to be interpreted within a functionally defined motor hierarchy.

We hypothesize that in chronic stroke, the canonical timing relationship between EEG and BOLD signals is altered, such that cross-modal alignment shows a shifted lag structure and cross-modal timing changes during paretic-limb stimulation. Specifically, we expect neurovascular coupling to be reduced within the lesioned (ipsilesional) sensorimotor network during paretic stimulation, with relatively greater coupling expressed in contralesional premotor and supplementary motor regions consistent with post-stroke hemispheric reweighting. We further hypothesize that coupling strength within ipsilesional motor territories will track motor impairment severity, providing a clinically interpretable index of neurovascular integrity. To test these hypotheses, we quantified how EEG-derived low-frequency oscillatory activity couples to BOLD responses during peripheral transcutaneous index-finger stimulation in chronic stroke participants and healthy controls. Unlike fixed bandpass approaches, which impose predefined frequency boundaries, Empirical Mode Decomposition (EMD) isolates subject-specific intrinsic oscillatory modes, enabling physiologically adaptive extraction of low-frequency envelopes for cross-modal timing analysis. EMD decomposed EEG signals into intrinsic mode functions (IMFs), from which physiologically relevant low-frequency components were isolated and Hilbert-transformed to obtain instantaneous amplitude envelopes. These LFO envelopes were used to model voxelwise BOLD fluctuations and to estimate cross-modal timing. This approach therefore quantifies regional, task-dependent cross-modal timing rather than global hemodynamic latency, allowing spatially resolved assessment of neurovascular temporal fidelity across the motor hierarchy. Because LFO envelopes are quasi-periodic, negative peaks in cross-correlation can arise as phase-wrapped equivalents when hemodynamic responses are substantially delayed; therefore, we interpret negative cross-correlation peaks as altered temporal alignment consistent with delayed hemodynamics, not as proof of reversed neurovascular causality (Mallat, 1999, Khan and Gotman, May 2003, Amiri Golilarz et al., 2020) . Spatial and hemispheric asymmetries in coupling were anatomically anchored using the Human Motor Area Template (HMAT), enabling voxelwise results to be interpreted within a validated motor hierarchy. Finally, we related coupling strength to clinical motor status using Fugl–Meyer Assessment (FMA) upper-extremity scores. By integrating EEG’s temporal resolution with fMRI’s spatial specificity, this framework enables region-resolved characterization of neurovascular timing across motor subregions and examines its relationship to motor impairment.

## 2 Methods

### 2.1 Participants, clinical characterization and stimulation paradigm

Thirteen participants were enrolled in this study, including six neurologically healthy controls and seven individuals with chronic unilateral ischemic stroke. Stroke participants were included based on a confirmed focal ischemic lesion and persistent upper-extremity motor impairment. Participant characteristics, including lesion laterality, age, sex, lesion volume, and clinical Fugl-Meyer Assessment (FMA; 0–66) scores, are summarized in Table 1. All participants underwent simultaneous EEG–fMRI acquisition during peripheral transcutaneous electrical stimulation (PTES) applied to the index finger in a block design (60 s stimulation, 60 s rest). Pulse duration was 200 µs with a 500 ms inter-stimulus interval.

**Table 1:** Participant demographics and clinical characteristics. Age and sex are reported for all participants. Controls were age- and sex-matched at the group level. Lesion side, vascular territory, lesion volume, and upper-extremity Fugl–Meyer Assessment (FMA; 0–66) scores are reported for stroke participants. Stimulation thresholds represent 2 × subjective sensory threshold in milliamperes (mA). Abbreviations: MCA, Middle Cerebral Artery; PCA, Posterior Cerebral Artery; L, left hemisphere; R, right hemisphere; P, paretic side; NP, non-paretic side.

Subject	Type	Lesion Side	Sex	Age	FMA	Primary Vascular Territory	Secondary / Branch Involvement	Lesion Volume (mm^3^)	Stimulation Threshold (mA)	Time Since Stroke
C1	Control	N/A	M	56	N/A	N/A	N/A	N/A	3.1 (L); 3.3 (R)	N/A
C2	Control	N/A	M	56	N/A	N/A	N/A	N/A	7.6 (L); 7.2 (R)	N/A
C3	Control	N/A	F	59	N/A	N/A	N/A	N/A	3.0 (L); 3.4 (R)	N/A
C4	Control	N/A	F	52	N/A	N/A	N/A	N/A	5.8 (L); 6.2 (R)	N/A
C5	Control	N/A	M	38	N/A	N/A	N/A	N/A	4.1 (L); 4.3 (R)	N/A
C6	Control	N/A	F	69	N/A	N/A	N/A	N/A	5.2(L); 4.8(R)	N/A
S1	Stroke	R	M	58	41	Right MCA	Superior and inferior divisions	1208	14.4 (P); 3.0 (NP)	3 years
S2	Stroke	R	M	61	63	Right MCA	Superior division	1078	17.0 (P); 11.2 (NP)	2 years
S3	Stroke	L	F	66	11	Right PCA	Posterior and superior division	1729	6.4 (P); 3.0 (NP)	7 years
S4	Stroke	L	F	62	32	Right MCA	Superior division	4489	17.0 (P); 4.6 (NP)	6 months
S5	Stroke	L	F	37	54	Right MCA	Lenticulostriate	5021	2.5 (P); 4.0 (NP)	2 years
S6	Stroke	L	M	70	44	Right MCA	Superior and inferior divisions	5155	7.8 (P); 12.0 (NP)	4 years
S7	Stroke	R	F	54	30	Right MCA	Superior division	1266	6.0 (P); 7.0 (NP)	10 months

Stimulation intensity was individually titrated to twice the sensory detection threshold, defined as the minimal current at which participants consistently reported a clear, non-painful tingling sensation. Thresholds were determined separately for each hand. To minimize variability in afferent drive, stimulation was further adjusted to produce visible but non-fatiguing finger movement and consistent subjective perceptual reporting across runs. All stimulation was maintained below the pain threshold. Control participants underwent stimulation separately on both hands. Stroke participants completed two separate runs: stimulation of the paretic index finger and stimulation of the non-paretic index finger. Because stroke can alter somatosensory perception, sensory thresholds were determined independently for each limb to ensure comparable relative afferent drive.

Absolute current values were recorded and verified to fall within a comparable physiological range across participants. Mean and standard deviation of stimulation current were 4.8±1.6mA in controls and 8.3±5.1mA for stroke participants (10.2±5.9mA during paretic-hand stimulation and 6.4±3.8mA during non-paretic hand stimulation). Delivered current ranges overlapped substantially across groups (controls: 3.0-7.6mA; stroke paretic:2.5-17.0mA; stroke non-paretic: 3.0-12.0mA), with no systematic between-group differences (p>0.05). Stroke participants were clinically stable at the time of testing. Information regarding vascular comorbidities and commonly prescribed medications (i.e., antihypertensives, statins, antiplatelet agents) was collected during screening, and while medication regimens were not experimentally controlled, all participants were tested in their usual medical state, and the effects of medication on vascular reactivity were not experimentally controlled. For group-level analyses, lesion laterality was normalized by flipping right-hemisphere lesions in MNI space such that the lesioned hemisphere was represented on the left. Accordingly, the stroke–paretic condition refers to stimulation of the hand contralateral to the lesioned hemisphere (probing ipsilesional cortex), whereas the stroke–non-paretic condition refers to stimulation ipsilateral to the lesion (probing contralesional cortex). The study was approved by the University of Oklahoma Institutional Review Board (IRB #12550), and all participants provided written informed consent).

Each stroke participant completed two stimulation runs: one with PTES applied to the paretic index finger and one with PTES applied to the non-paretic index finger, acquired in separate blocks. For group-level analyses, lesion side was normalized by flipping images of participants with right-hemisphere lesions in MNI space to the left, such that the lesioned hemisphere was represented on the left side of the image and the non-lesioned hemisphere on the right side for easy comparison and visualization. Accordingly, the stroke paretic condition refers to stimulation of the hand contralateral to the lesioned hemisphere (probing ipsilesional motor cortex), whereas the stroke non-paretic condition refers to stimulation of the hand ipsilateral to the lesion (probing the contralesional hemisphere).

### 2.2 Lesion segmentation and volume estimation

Stroke lesions were manually segmented on each participant’s T1-weighted anatomical image using ITK-SNAP (v4.4.0). Lesion masks were drawn in native anatomical space to preserve subject-specific lesion geometry and saved as binary NIfTI volumes. Lesion volume (mm3) was computed as:

$$V_{\text{lesion}}=N_{\text{vox}}\times V_{\text{vox}}$$

where $N_{\text{vox}}$ is the number of voxels labeled as lesion and $V_{\text{vox}}$ is voxel volume obtained from the T1 NIfTI header. Time since stroke at imaging ranged from 6 to 12 months (mean ± SD: 8.6 ± 2.4 months). All participants were in the chronic phase (>6 months post-stroke). Lesion laterality, lesion volume, and upper-extremity FMA scores are reported in Table 1. Lesions were visually classified according to vascular territory (i.e., middle cerebral artery distribution involving cortical and/or subcortical motor pathways). To facilitate anatomical interpretation and group-level visualization, lesion masks were transformed into MNI152 space and overlaid to generate a lesion overlap map (Supplementary Fig. S1). Importantly, volumetric estimates were computed in native space and not after spatial normalization.

### 2.3 EEG acquisition and preprocessing

EEG was recorded using a 64-channel MR-compatible BrainCap MR (Brain Products, GmbH, Gilching, Germany) conforming to the international 10–20 system, at a sampling rate of 1000 Hz. Electrode impedances were maintained below 10 kΩ. An ECG channel was recorded for cardiac artifact correction. Gradient artifacts induced by concurrent fMRI acquisition were removed using the FMRIB plugin within EEGLAB, which aligns EEG data to fMRI slice triggers and applies template subtraction. Residual gradient artifacts were further reduced using principal component analysis (PCA), with the first four components removed per segment. Subsequent processing was performed in MNE-Python (Gramfort, 2014). Ocular and muscle artifacts were removed using ICA, and cardiac contamination was minimized via regression of the ECG reference channel. To preserve ultra-low-frequency activity relevant to neurovascular coupling, no high-pass filter was applied. Signals were low-pass filtered at 40 Hz using a zero-phase 4th-order Butterworth filter. Line noise was attenuated using a 60 Hz notch filter. Cleaned signals were re-referenced to the common average and z-scored within participant prior to low-frequency oscillation (LFO) extraction (Delorme and Makeig, Mar. 2004).

### 2.4 MRI/fMRI acquisition and preprocessing

MRI data were acquired on a 3 T GE Discovery MR750 scanner using a 32-channel head coil. High-resolution T1-weighted anatomical images were obtained for structural localization and lesion segmentation (1 mm isotropic resolution). Functional images were acquired using a gradient-echo echo-planar imaging (EPI) sequence sensitive to BOLD contrast (TR = 2000 ms; TE = 43.5 ms; flip angle = 90°; voxel size = 2.5 mm isotropic; slice thickness = 2.9 mm). Functional preprocessing was performed fMRIPrep integrating tools from ANTs, FSL, FreeSurfer. Preprocessing steps included slice-timing correction, rigid-body motion correction, co-registration of functional images to the individual T1-weighted anatomical image, and spatial normalization to MNI152 standard space using affine transformation. Functional images were transformed to MNI space using the derived registration parameters and spatially smoothed with a 6 mm FWHM Gaussian kernel (Holmes et al., Mar. 1998, Behzadi et al., Aug. 2007) . To minimize distortion during normalization in lesioned brains, lesion masks were excluded from cost-function optimization during anatomical-to-template registration. This approach reduces warping artifacts in damaged tissue and preserves anatomical fidelity in perilesional regions. Physiological and motion confounds were modeled using anatomical CompCor (aCompCor). The first five principal components from white matter and cerebrospinal fluid masks were included as nuisance regressors, along with six rigid-body motion parameters and their temporal derivatives. Confound regressors were sampled at the fMRI repetition time (TR) and regressed from each voxel time series using linear nuisance regression with polynomial detrending. Temporal high-pass filtering was applied at 0.01 Hz to remove slow drifts. Preprocessed BOLD time series were retained in MNI space for group-level analyses and ROI-based coupling estimation.

### 2.5 LFO extraction via empirical mode decomposition

To isolate ultra-low-frequency oscillatory components from EEG signals, empirical mode decomposition (EMD) was applied independently to each EEG channel. EMD decomposes a non-stationary time series into a set of intrinsic mode functions (IMFs), denoted as ${{\mathrm{IMF}}_{i}\left(t\right)}_{i=1}^{n}$ , where n varies by channel and participant. For each IMF, the analytic signal was computed using the Hilbert transform, from which instantaneous frequency was estimated. IMFs whose dominant instantaneous frequency fell within the 0.1–1.5 Hz range were retained to represent low-frequency oscillations (LFOs) relevant to neurovascular coupling mechanisms. The composite LFO signal for each channel was defined as:

(1) $$\mathrm{LF}O_{\mathrm{EEG}}\left(t\right)=\sum_{i\in B}IMF_{i}\left(t\right)$$

where $B=\{i:f_{i}\in \left[0.1,1.5\right]\;\text{Hz}\}$ denotes the set of IMFs whose dominant instantaneous frequency $f_{i}$ lies within the specified LFO band. The amplitude envelope of the LFO signal was computed as the magnitude of the analytic signal:

(2) $$A_{\mathrm{LFO}}\left(t\right)=|H\left\{LFO_{\mathrm{EEG}}\left(t\right)\right\}|$$

where H{·} denotes the Hilbert transform. To reduce high-frequency envelope fluctuations while preserving slow amplitude modulations, the LFO envelope was smoothed using a third-order Savitzky–Golay filter (window length = 200 samples). Given the 1000 Hz sampling rate, this window length preserves modulation frequencies well below 5 Hz and does not attenuate the targeted 0.1–1.5 Hz band. The resulting LFO amplitude envelope was z-normalized within each participant prior to EEG–BOLD modeling.

To ensure cross-subject comparability, IMF inclusion was determined using a standardized frequency-based selection criterion rather than a fixed IMF index. Specifically, IMFs were retained if their dominant instantaneous frequency (via Hilbert transform) fell within the predefined LFO band (0.1–1.5 Hz). Across participants, the median number of retained IMFs per channel was 1 (interquartile range: 1–2), with no systematic difference between stroke and control groups. This frequency-constrained selection approach minimizes inter-subject variability in IMF indexing and ensures consistent physiological interpretation of the extracted oscillatory components.

### 2.6 EEG-BOLD modeling framework

EEG-derived LFO amplitude envelopes were temporally resampled to match the fMRI repetition time (TR = 2 s) and used as neural input regressors within a voxel-wise general linear model (GLM). To assess robustness to assumptions regarding hemodynamic shape and latency, three complementary hemodynamic response function (HRF) modeling strategies were implemented: (i) canonical HRF convolution, (ii) derivative-augmented basis modeling, and (iii) subject-specific HRF estimation via deconvolution. Across all models, coupling magnitude, sign consistency, and temporal alignment metrics were evaluated to determine whether group-level effects depended on HRF assumptions.

#### 2.6.1 Canonical HRF model

In the canonical model, LFO envelopes were convolved with a double-gamma HRF as implemented in SPM. The canonical HRF models both the primary positive BOLD peak and post-stimulus undershoot using a physiologically constrained impulse response.

Let x(t) denote the EEG-derived LFO envelope and $h_{\mathrm{can}}\left(t\right)$ the canonical HRF. The predicted BOLD response was defined as:

(3) $$\hat{y}\left(t\right)=\left(x*h_{\mathrm{can}}\right)\left(t\right)$$

where ∗ denotes convolution. The convolved regressor was variance-normalized prior to inclusion in the GLM. Voxel-wise beta weights were estimated using ordinary least squares. Statistical maps were converted to Fisher z-values for group-level analyses.

#### 2.6.2 Derivative-augmented HRF basis set

To accommodate inter-individual variability in HRF latency and dispersion, a derivative-augmented basis set was implemented. In addition to the canonical HRF, its temporal derivative and dispersion derivative were included as regressors. This basis expansion allows local adjustments in peak timing and width while preserving physiologically plausible HRF morphology. The model therefore takes the form:

(4) $$\hat{y}\left(t\right)=\beta_{1}\left(x*h_{\mathrm{can}}\right)\left(t\right)+\beta_{2}\left(x*h_{\mathrm{temp}}\right)\left(t\right)+\beta_{3}\left(x*h_{\mathrm{disp}}\right)\left(t\right)$$

where $h_{\mathrm{temp}}\left(t\right)$ and $h_{\mathrm{disp}}\left(t\right)$ denote the temporal and dispersion derivatives of the canonical HRF.

Statistical inference was based on the joint contribution of canonical and derivative terms. This approach tests whether group differences reflect simple latency shifts rather than altered coupling polarity.

#### 2.6.3 Subject-specific HRF estimation

To further minimize dependence on fixed HRF assumptions, subject-specific hemodynamic response functions were estimated using regularized deconvolution.

Given the EEG-derived neural input x(t) and observed BOLD signal y(t), the impulse response h(t) was estimated under the linear convolution model:

(5) y(t)=(x∗h)(t)+∊(t)

where ∊(t) denotes residual error. HRFs were constrained to finite temporal support (0–20 s) and smoothness using Tikhonov regularization to prevent overfitting. Estimated HRFs were then used to reconstruct predicted BOLD responses for each subject. Coupling magnitude and temporal alignment metrics were recomputed using these individualized impulse responses. Consistency of coupling sign, magnitude ordering, and lag polarity across canonical, derivative-augmented, and subject-specific models was used to assess robustness of findings to HRF specification.

### 2.7 Temporal alignment analysis

#### 2.7.1 Cross-Correlation lag Estimation, peak selection and Phase-Wrapping control

Temporal alignment between EEG-derived LFO envelopes and BOLD time series was quantified using cross-correlation within a ±10s lag window:

(6) $$\tau^{*}=arg\underset{\tau \in [-10,10]}{max}\text{Corr}\left(LFO_{\mathrm{EEG}}\left(t\right),BOLD\left(t+\tau \right)\right)$$

The lag τ∗ corresponding to the maximal correlation was defined as the peak alignment lag. Positive values indicate EEG-leading BOLD dynamics; negative values indicate apparent BOLD-leading dynamics. Because LFO signals exhibit quasi-periodic structure (0.1Hz;period≈10s), cross-correlation peaks may recur at intervals equal to the oscillatory period. To mitigate phase-wrapping ambiguity, a physiology-constrained lag estimate (τ_phys) was computed by restricting plausible neurovascular delays to 0-8s (EEG-leading). For negative τ∗ values occurring near -T_LFO/2, the equivalent positive peak was identified by adding one oscillatory period:

(7) $$\tau_{\mathrm{phys}}=\tau^{*}+T_{\mathrm{LFO}}$$

where $T_{\mathrm{LFO}}\approx 10$ s. Both raw τ∗ and τ_phys were retained for analysis. The proportion of negative τ∗ values was reported descriptively to quantify phase ambiguity.

#### 2.7.2 Event-locked peak timing analysis

Event-locked BOLD responses were extracted from HMAT-constrained sensorimotor ROIs and aligned to stimulation onset. For each subject and condition, ROI-averaged BOLD time courses were baseline-corrected using the pre-stimulus interval (-10to0s) and averaged across stimulation blocks within-run to obtain a single event-locked response. To reduce noise-driven local maxima, time courses were temporally smoothed (Gaussian kernel; σ=1TR). Peak latency was defined as the time-to-maximum percent signal change within a post-stimulus window selected to capture the hemodynamic peak under both control and delayed-stroke responses (i.e., 0-40s; Fig. 3). This peak-latency measure provides a model-agnostic estimate of hemodynamic timing and was used to contextualize cross-correlation–based lag estimates. When the raw cross-correlation peak lag (τ∗) was negative, delayed event-locked peak latencies were interpreted as supporting phase-wrapping ambiguity under prolonged hemodynamic delay, rather than evidence of true BOLD-leading neurophysiology. A physiology-constrained lag estimate (τ_phys) was therefore derived by mapping phase-equivalent peaks into a plausible EEG → BOLD delay range.

### 2.8 Spatial ROI definition, HMAT quantification, and hemispheric asymmetry

Voxel-wise EEG–BOLD coupling maps (Fisher z-transformed) were spatially normalized to MNI152 space for group-level visualization and region-based quantification. To enable hemispheric comparisons independent of lesion laterality, stroke participants with right-hemisphere lesions were left–right flipped in MNI space such that the lesioned hemisphere was consistently represented on the left (ipsilesional) and the non-lesioned hemisphere on the right (contralesional). For control participants, hemispheres were defined relative to the stimulated hand (contralateral vs. ipsilateral). To anatomically constrain coupling estimates to motor-relevant cortex, coupling maps were masked using the Human Motor Area Template (HMAT). ROI-level coupling strength was computed for each participant and condition by averaging Fisher z-values within HMAT parcels: primary motor cortex (M1), primary somatosensory cortex (S1), dorsal premotor cortex (PMd), ventral premotor cortex (PMv), supplementary motor area (SMA), and pre-SMA. ROI summaries were computed separately for each stimulation condition (controls: left-hand and right-hand; stroke: paretic-hand and non-paretic-hand stimulation). Group-level HMAT maps were generated by averaging normalized coupling maps within each group/condition using identical masking and visualization parameters. Hemispheric asymmetry in coupling was quantified using a laterality index (LI):

(8) $$\mathrm{LI}=\frac{C-I}{C+I}$$

where C and I denote mean HMAT-constrained coupling strength (Fisher z) in contralateral and ipsilateral hemispheres, respectively (controls: relative to stimulated hand; stroke: relative to normalized lesion side—contralesional vs. ipsilesional). Positive LI values indicate greater coupling in the hemisphere contralateral to the stimulated hand. Negative LI values indicate relatively greater coupling in the ipsilateral hemisphere. For stroke participants, hemispheres were interpreted within normalized lesion space (ipsilesional = left; contralesional = right), allowing condition-specific interpretation without altering LI sign convention.

### 2.9 HMAT ROI quantification, stroke subgroup definition, and clinical correlation

Voxel-wise EEG–BOLD coupling maps were converted to Fisher z-values and summarized within motor-system regions defined by the Human Motor Area Template (HMAT). For each participant and condition, mean coupling strength was computed by averaging z-values within HMAT parcels (M1, S1, PMd, PMv, SMA, pre-SMA). For stroke participants, all maps were transformed to a normalized ipsilesional/contralesional coordinate system by flipping right-hemisphere lesions to the left in MNI space, enabling hemispheric comparisons independent of lesion laterality. To visualize recovery-linked differences, stroke participants were stratified by upper-extremity Fugl–Meyer Assessment (FMA) using clinically motivated thresholds: low FMA (<35; moderate-to-severe impairment) and high FMA (≥45; mild impairment). Participants with intermediate scores (35–44) were excluded from subgroup visualizations to preserve categorical separation; subgroup analyses were interpreted descriptively due to small sample size. Behavioral relevance was assessed by correlating FMA scores with (i) mean HMAT ROI coupling strength and (ii) physiology-constrained timing metrics (τ_phys and/or event-locked peak latency summaries). Pearson correlations and linear regression were used to estimate association strength and 95% confidence intervals (two-tailed α = 0.05).

### 2.10 Statistical analysis

Analyses tested group differences in neurovascular timing, coupling strength, hemispheric asymmetry, and clinical association. For temporal alignment, peak cross-correlation lag was estimated within ±10s and converted to a physiology-constrained lag (τ_phys) by restricting plausible neurovascular delays to an EEG-leading window (0-8s) and correcting phase-wrapped negative peaks by addition of one LFO period where applicable. Group differences in τ_phys were assessed using independent two-sample t-tests; the proportion of negative raw τ∗ values were summarized descriptively as an index of phase-wrapping ambiguity. Event-locked peak latency metrics were summarized to provide an independent timing check. For coupling strength, voxel-wise Fisher z-maps were evaluated with correction for multiple comparisons using FDR (Benjamini–Hochberg, q<0.05). ROI-level coupling values within HMAT parcels were analyzed using mixed-effects ANOVA with Group (Control vs Stroke) as a between-subject factor and Hemisphere (ipsilesional vs contralesional; defined relative to stimulation/lesion conventions) as a within-subject factor. ROI post hoc tests were corrected using Holm–Bonferroni. Hemispheric asymmetry was quantified using the laterality index (LI) and compared using independent t-tests. Clinical association was evaluated using Pearson correlation and linear regression between FMA scores and HMAT ROI coupling strength and timing metrics (τ_phys and/or peak latency summaries). Effect sizes are reported as Cohen’s d (pairwise) and partial η2 (ANOVA). All tests were two-tailed with α=0.05.

## 3 Results

Stroke altered the timing, magnitude, and spatial organization of neurovascular responses during peripheral finger stimulation. We first summarize lesion anatomy across the stroke cohort, then quantify stimulation-evoked BOLD timing and amplitude. We next test whether stroke-related delays can be explained by modest HRF variability (informed basis set) versus more substantial hemodynamic slowing. These results motivate subsequent cross-modal timing analyses of LFO–BOLD alignment using physiology-constrained lag estimation.

### 3.1 Lesion distribution across the stroke cohort

Fig. 2 demonstrates heterogeneous infarct extent with convergent involvement of front-to-parietal sensorimotor territories relevant to index-finger stimulation. Infarcts frequently involved *peri*-rolandic cortex and/or adjacent subcortical white matter, with variable extension into premotor and parietal regions.

**Fig. 1:**
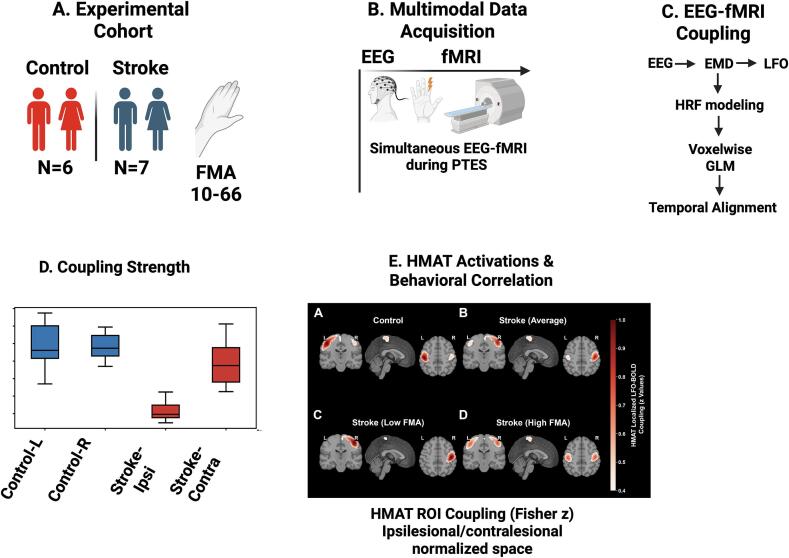
Study design and analysis framework for LFO–BOLD coupling in chronic stroke. (A) Cohort composition: controls and chronic stroke participants (Fugl–Meyer Assessment range 10–66). (B) Simultaneous EEG–fMRI acquisition during peripheral transcutaneous electrical stimulation (PTES) of the index finger using a block design. Controls were stimulated on both hands; stroke participants completed paretic and non-paretic runs. (C) EEG–fMRI coupling pipeline. EEG signals were decomposed using empirical mode decomposition (EMD) to isolate low-frequency oscillations (LFOs). LFO envelopes were entered into voxelwise GLM analyses using three hemodynamic modeling strategies (canonical HRF, derivative-augmented informed basis set, and subject-specific HRF estimation). Temporal alignment between EEG-derived LFO activity and BOLD fluctuations was quantified using cross-correlation lag estimation with physiology-constrained phase-wrapping control and complementary event-locked peak analyses. (D) Group and condition comparisons of HMAT-constrained LFO–BOLD coupling strength. (E) Spatial localization of coupling within Human Motor Area Template (HMAT) ROI in ipsilesional/contralesional normalized space.

**Fig. 2:**
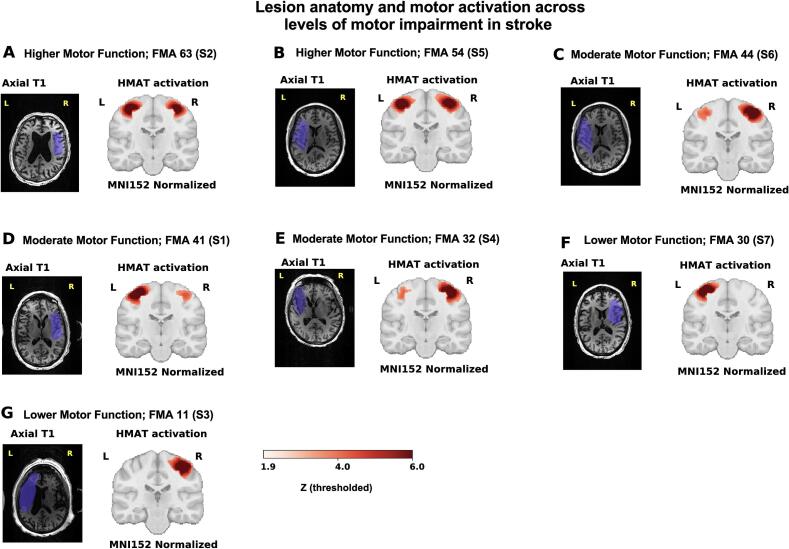
Lesion anatomy across stroke participants stratified by motor impairment. Axial T1-weighted images (MNI152 normalized space) show individual infarcts (purple) across the stroke cohort, ordered by Fugl–Meyer motor score (FMA). Lesions predominantly involve *peri*-Rolandic cortex and adjacent subcortical white matter, with variable extension into dorsal premotor and parietal territories. Right panels show HMAT-constrained sensorimotor activation maps during paretic-hand stimulation for the same participants. Despite heterogeneity in lesion extent, infarcts frequently involve *peri*-Rolandic and adjacent subcortical regions relevant to the stimulation paradigm. (For interpretation of the references to colour in this figure legend, the reader is referred to the web version of this article.)

### 3.2 Stroke delays and attenuates the stimulation-evoked BOLD response

**Fig. 3:**
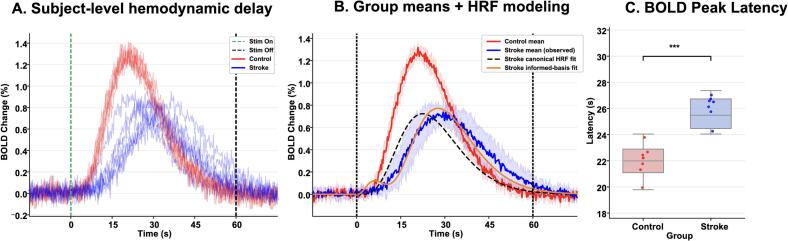
Stroke delays and attenuates the stimulation-evoked BOLD response. (A) Event-locked BOLD responses averaged within HMAT-constrained sensorimotor ROIs and aligned to stimulation onset. Vertical dashed lines indicate stimulation onset and offset. (B) Group mean BOLD responses with canonical HRF and derivative-augmented informed basis set fits. In stroke participants, peak timing is delayed relative to controls, and derivative modeling improves waveform alignment but does not fully normalize timing. (C) Subject-level peak latency extracted from ROI-averaged time courses. Stroke participants exhibit significantly prolonged BOLD peak latency compared with controls (∗∗∗p<0.001).

Event-locked BOLD responses time-locked to stimulation onset demonstrated both slower peak timing and reduced response magnitude in stroke relative to controls (Fig. 3). In controls, peak BOLD occurred at 11.6±1.8s, whereas stroke participants showed a significant delay (16.4±2.1s, $t\left(11\right)=6.27,p=4.8\times 10^{-4}$). Peak percent signal change was also reduced in stroke (0.71%±0.12) compared with controls (1.14%±0.15,p<0.01).

### 3.3 Event-locked HRF modeling confirms impairment-linked hemodynamic delay

**Fig. 4:**
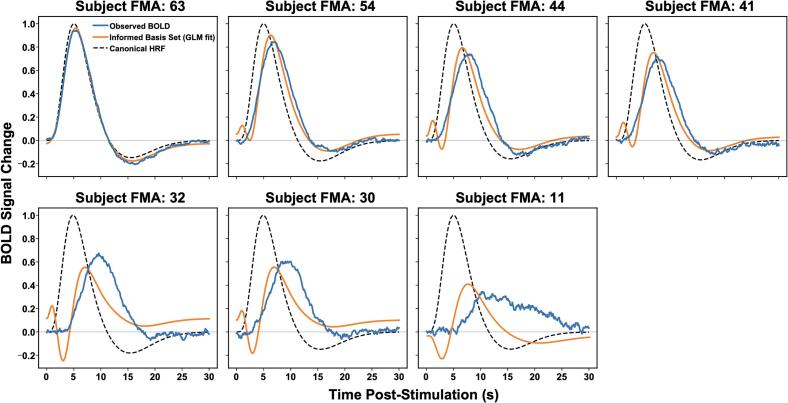
Per-subject event-locked BOLD responses and HRF model fits ranked by motor impairment. Observed ROI-averaged BOLD responses (blue) are shown alongside canonical HRF fits (black dashed) and derivative-augmented informed-basis fits (orange). Subjects are ordered by decreasing Fugl–Meyer motor score. In lower-FMA participants, BOLD responses exhibit delayed peak timing and prolonged dispersion. Informed-basis modeling improves waveform alignment relative to the canonical HRF but does not fully normalize peak timing in more impaired individuals.. (For interpretation of the references to colour in this figure legend, the reader is referred to the web version of this article.)

To distinguish modest HRF variability from more substantive hemodynamic slowing, event-locked BOLD responses were fit using (i) a canonical HRF and (ii) a derivative-augmented informed basis set including temporal and dispersion derivatives (Fig. 4). Canonical HRF fits showed systematic peak timing mismatch in stroke, with larger peak latency errors in more impaired participants. Canonical HRF models systematically underestimated peak latency in stroke, with error magnitude scaling with motor impairment (Supplementary Fig. S1; Supplementary Table S1). Allowing temporal/dispersion flexibility improved model recovery and reduced peak timing error relative to the canonical model; however, residual delay persisted in more impaired participants, consistent with stroke-related slowing beyond minor dispersion differences. Derivative-augmented models improved cross-validated generalization in 6 of 7 S participants (Supplementary Fig. S2; Supplementary Table S1). Per-subject peak timing, timing error, and cross-validated model recovery metrics are provided in Supplementary Table S1.

### 3.4 Stroke disrupts spatial localization of LFO–BOLD coupling in a condition-dependent manner

**Fig. 5:**
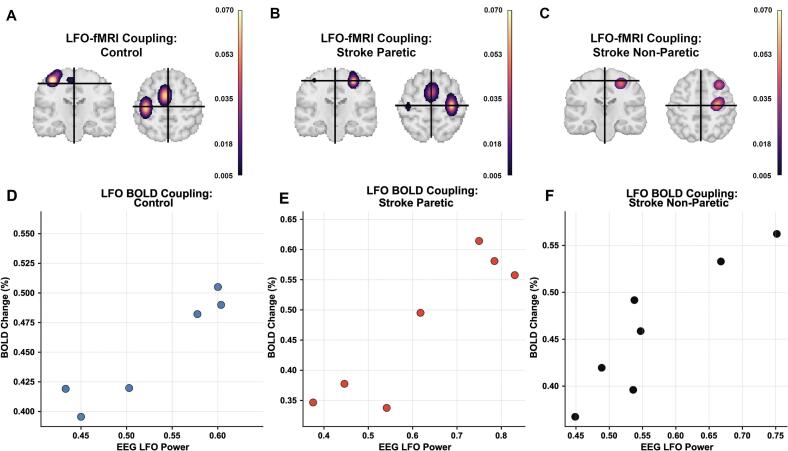
Condition dependent alterations in ROI-level LFO-BOLD coupling after stroke. (A–C) Group-level voxelwise LFO–fMRI coupling maps. Controls (A) exhibit focal contralateral sensorimotor coupling. During paretic-hand stimulation in stroke (B), coupling is attenuated within ipsilesional motor cortex with relatively greater contralesional expression. Non-paretic stimulation (C) shows partial preservation of expected topographic organization. (D–F) ROI-level relationships between EEG LFO power and task-evoked BOLD percent signal change for the corresponding conditions. Each point represents one participant (mean across stimulation blocks). Controls demonstrate relatively consistent positive coupling. Stroke participants show greater dispersion during paretic stimulation, consistent with reduced neurovascular gain, while non-paretic stimulation exhibits intermediate variability.

Voxelwise LFO–BOLD coupling maps demonstrated focal contralateral sensorimotor localization in controls, with peak coupling within canonical motor territories (M1/S1 and SMA; Fig. 5A). During paretic-hand stimulation in stroke participants, coupling was attenuated within ipsilesional cortex and appeared spatially less cohesive, with relatively greater contralesional expression (Fig. 5B). In contrast, non-paretic stimulation showed partial preservation of expected topographic organization (Fig. 5C). ROI-level analysis confirmed reduced coupling strength in stroke relative to controls (0.038±0.007 vs 0.057±0.008; p<.001), with the greatest reduction observed during paretic stimulation. These findings indicate a condition-dependent disruption of neurovascular alignment within the motor network following stroke.

### 3.5 Coupling strength scales with lesion-side stimulation and motor status

**Fig. 6:**
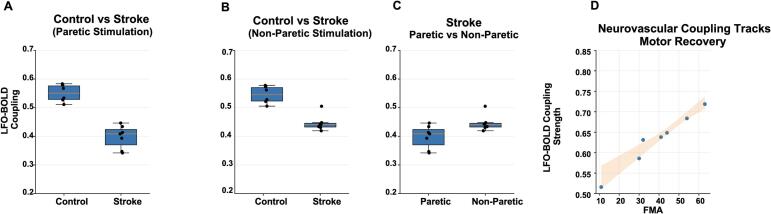
LFO–BOLD coupling strength differs by group, condition, and impairment severity. (A) Controls show higher coupling than stroke during paretic-hand stimulation. (B) Coupling remains reduced in stroke during non-paretic stimulation. (C) Within stroke, paretic stimulation yields lower coupling than non-paretic stimulation (paired lines show within-subject change). (D) In stroke participants, paretic-side coupling strength increases with upper-extremity Fugl–Meyer score (shaded band: 95% CI).

Within the stroke cohort, paretic-hand stimulation elicited lower coupling strength than non-paretic stimulation (p=0.03; Fig. 6C), indicating lesion-side specificity. Importantly, paretic-side coupling strength positively correlated with upper-extremity motor function Fig. 6D. Given the modest stroke cohort (n = 7), this association should be interpreted as hypothesis-generating, and sensitivity to individual data points is expected; replication in a larger sample will be necessary to determine its stability and predictive value.

### 3.6 Temporal dynamics and hemispheric reorganization of LFO-BOLD coupling in chronic stroke

**Fig. 7:**
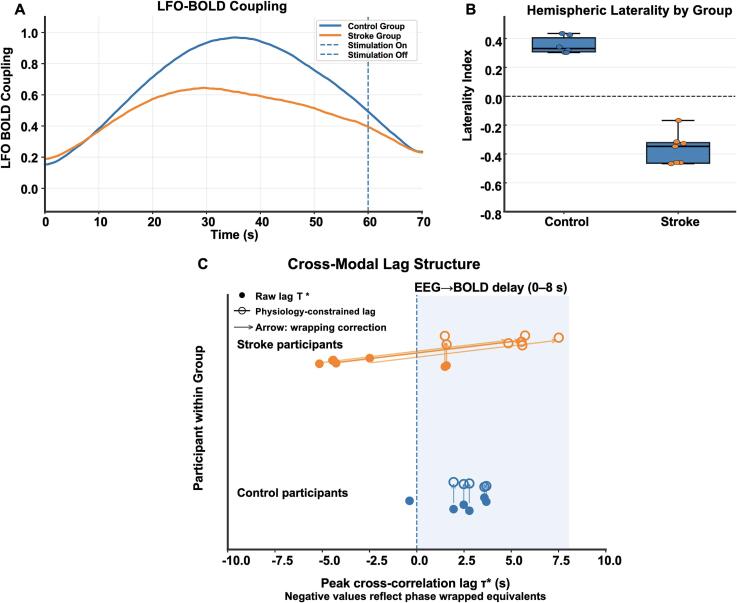
Temporal dynamics, hemispheric reorganization, and cross-modal lag structure of LFO–BOLD coupling in chronic stroke. (A) Time-resolved LFO–BOLD coupling across the stimulation block. Controls show a stimulus-locked rise after stimulation onset, sustained mid-block elevation, and a return toward baseline after stimulation offset, consistent with stable stimulus-driven neurovascular coordination. Stroke participants show reduced coupling magnitude and a compressed dynamic range, indicating diminished ability to sustain coordinated neural–hemodynamic alignment during stimulation. Vertical dashed lines denote stimulation onset and offset. (B) Hemispheric balance quantified using the lateralization index (LI) computed from HMAT-constrained coupling values (contralateral vs ipsilateral to the stimulated hand; in stroke, reported in normalized ipsilesional/contralesional space; Methods). Controls exhibit consistent contralateral dominance (LI>0). Stroke participants shift toward contralesional weighting (LI<0) with greater inter-subject variability, consistent with lesion-side suppression and compensatory recruitment. (C) Peak-lag estimates from cross-correlation between EEG-derived LFO envelopes and BOLD time series within ±10s. Filled markers denote the raw peak lag (τ∗). For participants with negative τ∗ values, open markers indicate the physiology-constrained lag (τ_phys) obtained by mapping phase-wrapped equivalents into a plausible EEG → BOLD delay range (0-8s; shaded region). Negative τ∗ values are interpreted as phase-wrapped alignment under delayed hemodynamics rather than proof of reversed neurovascular causality. Connecting lines indicate within-subject mapping from τ∗ to τ_phys.

Time-resolved coupling analyses showed that stimulus-locked LFO–BOLD coupling evolves over the course of the stimulation block and differs by group (Fig. 7A). Controls exhibited a coherent increase in coupling following stimulation onset, a sustained mid-block elevation, and a return toward baseline after stimulation offset, consistent with stable neural–hemodynamic coordination during ongoing afferent drive. In stroke, coupling magnitude was reduced and dynamic range was compressed, indicating diminished ability to sustain coordinated neural–vascular alignment during stimulation. Hemispheric balance analyses further demonstrated reorganization of coupling lateralization after stroke (Fig. 7B). Controls showed consistent contralateral dominance across stimulation conditions, whereas stroke participants exhibited reduced ipsilesional dominance with a shift toward contralesional weighting and increased inter-subject variability. This hemispheric reweighting is consistent with lesion-side suppression of ipsilesional sensorimotor coupling and compensatory contralesional recruitment observed in HMAT-localized maps (Section 3.8).

### 3.7 Cross-modal lag structure is shifted after stroke but is consistent with delayed hemodynamics rather than reversed causality

Peak cross-correlation lag (τ∗) between EEG-derived LFO envelopes and ROI-averaged BOLD time series differed systematically by group. Controls exhibited predominantly positive τ∗ values, consistent with canonical EEG-leading neurovascular timing. Stroke participants demonstrated reduced and frequently negative τ∗ values, suggesting altered temporal alignment. Because the LFO envelope is quasi-periodic (0.1 Hz; period ≈ 10s), cross-correlation maxima recur at phase-equivalent offsets. When the true hemodynamic delay approaches a substantial fraction of the oscillatory period, the dominant correlation peak may shift into the negative-lag window due to phase wrapping rather than physiological inversion. Consistent with this mechanism, event-locked analyses (3.2, 3.3) demonstrated prolonged hemodynamic latency in stroke. Applying a physiology-constrained lag estimate (τ_phys), restricting EEG-leading delays to 0-8s, mapped all negative τ∗ values to positive phase-equivalent delays (Supplementary Fig. S3; Supplementary Table S2). Even under this constraint, stroke participants exhibited significantly prolonged τ_phys relative to controls.

### 3.8 Hmat-localized network reorganization across recovery levels

**Fig. 8:**
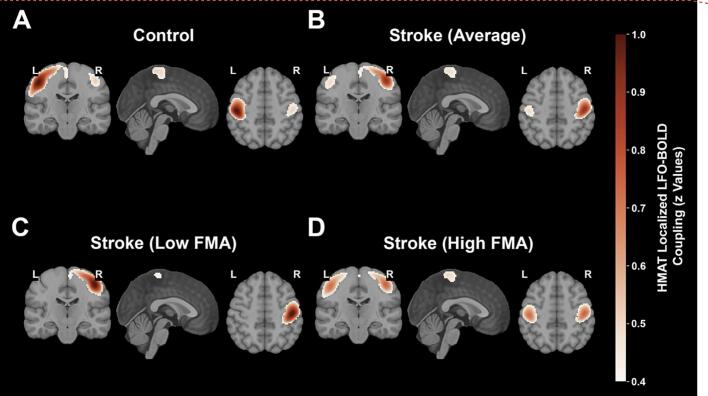
HMAT-localized LFO-BOLD coupling maps in normalized ipsilesional/contralesional space. Group-averaged voxelwise coupling maps (Fisher z-transformed) are shown after anatomical restriction to motor territories. For stroke participants, right-hemisphere lesions were flipped to a common orientation such that the lesioned hemisphere is displayed on the left (ipsilesional) and the intact hemisphere on the right (contralesional). (A) Controls show focal contralateral coupling in canonical motor territories. (B) Stroke (all participants) shows reduced ipsilesional coupling with comparatively greater contralesional expression. (C) Low-FMA subgroup exhibits the most pronounced ipsilesional suppression and hemispheric imbalance. (D) High-FMA subgroup shows more bilateral coupling patterns relative to low-FMA. Scale denotes HMAT-localized coupling strength (Fisher z) in MNI space.

HMAT parcellation localized group differences to canonical motor territories, including M1, PMd, and SMA (Fig. 8). Controls showed focal contralateral coupling within HMAT motor regions. Stroke participants exhibited reduced ipsilesional coupling with relatively greater contralesional expression, consistent with hemispheric reweighting during paretic-hand stimulation. When visualized by recovery level, low-FMA participants demonstrated the strongest ipsilesional suppression and hemispheric imbalance, whereas high-FMA participants showed partially restored and more bilateral coupling patterns. These subgroup visualizations are descriptive given the limited sample size, but they illustrate systematic heterogeneity in coupling topology that tracks motor impairment severity.

## 4 Discussion

Neurovascular coupling is typically described as a feed-forward process in which neural activity precedes and drives delayed hemodynamic responses and in the healthy brain, this relationship is robust and predictable; however, in the context of stroke, vascular reactivity is known to be both attenuated and temporally altered (Phillips et al., Nov. 2015, Iadecola, 2017, Girouard and Iadecola, 2006) . Understanding how these pathological changes reshape the alignment between electrophysiological dynamics and BOLD responses during active sensorimotor engagement remains incompletely defined. The present study addressed this question using simultaneous EEG–fMRI during peripheral transcutaneous electrical stimulation, analyzed within a multimodal framework (Fig. 1). Across multiple analytic levels, a coherent pattern emerged: event-locked responses demonstrate delayed and attenuated hemodynamics in stroke (Fig. 3), while spatial maps show reduced ipsilesional coupling during paretic stimulation with a corresponding hemispheric redistribution toward contralesional territories (Fig. 3, Fig. 4). Furthermore, coupling strength varies with motor status (Fig. 5), and the cross-modal lag structure reflects a delayed temporal alignment rather than inversion of physiological directionality (Fig. 7). Together, these findings indicate altered neurovascular timing and hemispheric reweighting. The most prominent physiological signal in this dataset is the significant event-locked delay observed in the stroke cohort. In this context, our results support a loss of neurovascular temporal fidelity in the ipsilesional motor network, characterized by delayed and spatially heterogeneous alignment between neuronal oscillatory envelopes and hemodynamic responses. Individual and group-mean BOLD time courses (Fig. 2A–B) show reduced amplitude and visibly delayed peak responses in stroke participants relative to healthy controls. Quantitative analysis of these peaks confirms a substantial latency shift (Fig. 2C), and group modeling (Fig. 6A–C) demonstrates that this delay persists even after derivative-augmented informed basis fitting.

This persistence is a critical observation indicating that the slowing associated with stroke exceeds the range of ordinary inter-individual hemodynamic response function (HRF) variability (Rangaprakash et al., Oct. 2018, Steffener et al., Feb. 2010, Aguirre et al., Nov. 1998) . Furthermore, this observation directly constrains the interpretation of cross-modal lag estimates, since the BOLD response is delayed by several seconds relative to the neural trigger, any cross-correlation metrics computed on continuous oscillatory signals will necessarily reflect a shifted delay structure. These findings align with established literature documenting impaired cerebrovascular reactivity, altered autoregulation, and microvascular remodeling following ischemic or hemorrhagic injury (Hu et al., Feb. 2017, “Chapter 14 Cerebral vascular dysregulation in the ischemic brain,”, 2008, Girouard and Iadecola, 2006) . While delayed BOLD responses have been documented in both acute and chronic phases of stroke, they are generally interpreted as reflecting altered neurovascular transfer dynamics rather than a delay in the neural onset itself (Altamura, Dec. 2009, Mazzetto-Betti, Sep. 2010) . Our data extends this framework by demonstrating that hemodynamic slowing alters multimodal temporal alignment, and this temporal delay is accompanied by a structured spatial reorganization within the motor hierarchy. Particularly, in healthy controls, EEG-informed voxelwise maps localize coupling primarily to the contralateral sensorimotor cortex during unilateral stimulation (Fig. 3A), with ROI-level confirmation in M1, PMd, and the SMA (Fig. 4A–C). Hemispheric laterality indices in the control group show consistent contralateral dominance, as expected (Fig. 4B). In contrast, paretic-hand stimulation in the stroke group produces reduced and spatially fragmented coupling within the ipsilesional M1 and PMd (Fig. 3B), with relatively greater expression in contralesional premotor and supplementary motor areas (Fig. 8B–C). Importantly, these effects are anatomically structured within HMAT-defined motor territories, which argues against a purely systemic vascular artifact or global noise. Further, we interpret this as a redistribution of coupling expression consistent with contralesional recruitment described in prior neurorehabilitation studies (Bestmann, Sep. 2010, Johansen-Berg et al., Oct. 2002, Thickbroom, Aug. 2015) . Whether this recruitment represents a compensatory mechanism or a maladaptive consequence of reduced ipsilesional inhibition remains a subject of debate in the field; however, our results provide a clear multimodal signature of this hemispheric reweighting. The cross-modal lag structure observed in this study provides a specific cautionary note regarding the interpretation of coupling direction. In controls, raw cross-correlation lags τ∗ clustered in the expected EEG-leading range (Fig. 7C).

However, in stroke participants, τ∗ values shifted toward smaller and occasionally negative values. A literal interpretation of negative lags would suggest that BOLD activity leads neural activity, which would violate the fundamental principles of the neurovascular unit. However, the EEG-derived LFO envelope exhibits a quasi-periodic structure within the 0.1–1.5 Hz band. In cross-correlation analysis, peaks recur at offsets separated by approximately one oscillatory period. When the observed hemodynamic delay is significant, i.e., approaching a substantial fraction of that oscillatory period, the dominant correlation maximum can shift into the negative-lag window through phase wrapping or aliasing. Beyond the mean delay, the increased dispersion of lag estimates in the stroke cohort suggests reduced temporal stability of cross-modal alignment, consistent with heterogeneous neurovascular transfer dynamics across motor regions. Thus, by comparing raw τ∗ and physiology-constrained τ_phys values (Fig. 7C), we demonstrate that the observed lag shifts reflect altered temporal alignment under oscillatory ambiguity, rather than a reversal of neurovascular directionality. This highlights the necessity of anchoring continuous correlation metrics to independent event-locked observations when studying pathological populations. Within the stroke cohort, the strength of paretic-side coupling correlated positively with Fugl–Meyer motor scores (Fig. 5D). Participants with lower FMA scores exhibited more pronounced ipsilesional suppression and greater contralesional weighting (Fig. 8C), whereas higher-functioning participants showed a partial restoration of bilateral engagement (Fig. 8D). Given the modest sample size (n=7), these behavioral associations are exploratory. The convergence of delayed hemodynamics, reduced ipsilesional coupling, and motor impairment is consistent with the hypothesis that neurovascular alignment reflects motor network integrity. Future investigations should expand upon these findings by incorporating a wider range of clinical covariates. Factors such as the use of antihypertensives, statins, or anticoagulants, as well as the presence of vascular comorbidities like hypertension and diabetes, are known to influence cerebrovascular reactivity and should be explicitly modeled in larger cohorts to isolate the specific effects of the stroke lesion from general vascular health. Furthermore, while participants were clinically stable on standard secondary stroke prevention regimens (i.e., statins, antihypertensives), the potential for these agents to modulate neurovascular coupling cannot be entirely excluded. However, given that participants served as their own internal controls (paretic vs. non-paretic hand), the impact of systemic medication on the observed hemispheric asymmetries is likely minimized. Several methodological considerations merit clarification to ensure the robustness of these findings. First, the 0.1–1.5 Hz LFO band partially overlaps with cardiac harmonics. To mitigate this, we utilized ECG regression, ICA-based artifact removal, and the inclusion of motion and aCompCor components. The fact that our results are localized to specific HMAT motor territories and vary by condition (paretic vs. non-paretic) suggests that the findings are not the result of systemic physiological contamination.

Second, while Empirical Mode Decomposition (EMD) was used to adaptively separate slow trends, residual non-neural contributions to the LFO cannot be entirely ruled out. Third, lag estimation was confined to a 10 s window approximating one oscillatory cycle near 0.1 Hz and typical neurovascular delays; future work should assess the robustness of these estimates across alternative window widths to ensure that the chosen window does not bias the detection of pathological delays. Additionally, we acknowledge a potential methodological contradiction in using a canonical HRF framework to detect HRF pathology. While derivative-augmented basis fitting (Fig. 6) captures latency shifts, it remains constrained by a predefined physiological envelope. The present study does not establish causality between neural and vascular changes but instead characterizes their temporal alignment under controlled sensorimotor input and future studies might employ more “model-free” deconvolution approaches to characterize the shape of the stroke HRF without the constraints of a healthy-brain template. Furthermore, analysis with larger cohorts should incorporate detailed lesion volumes and vascular territory mapping to further refine the anatomical interpretation of coupling shifts. In conclusion, these findings suggest that chronic stroke reshapes neurovascular timing and spatial coupling expression without violating basic feed-forward physiology. The observed delays in the BOLD response introduce an inherent ambiguity in oscillatory lag estimation, which can be resolved through physiology-constrained interpretation. Furthermore, the resulting spatial redistribution of coupling localizes within anatomically defined motor territories and is consistent with the hypothesis that neurovascular alignment reflects motor network integrity.

A critical consideration in interpreting post-stroke neuroimaging data is the potential confound of differential afferent input during task performance. In the current study, absolute stimulation current values were maintained within comparable physiological ranges across all cohorts. While the stroke group exhibited higher mean thresholds on the paretic side (10.2±5.9mA) compared to controls (4.8±1.6mA), these differences did not reach statistical significance (p>0.05), and individual ranges showed substantial overlap (stroke paretic: 2.5-17.0mA and controls: 3.0-7.6mA).By individually titrating stimulation to twice the subjective sensory threshold, we sought to standardize the perceived salience of the stimulus. This approach is important to ensure that observed variations in BOLD activation patterns reflect true cortical reorganization or functional deficits rather than simple artifacts of stimulus magnitude. Although post-stroke alterations in sensory processing may introduce inherent variability in afferent drive, the lack of systematic group-level differences in delivered current suggests that our findings are not driven by biased stimulus intensity. Future research utilizing objective measures, such as somatosensory evoked potentials (SEPs), could further refine this control by quantifying the neural arrival of the afferent volley at the primary sensory cortex. However, future studies incorporating objective sensory or evoked-potential measures would further strengthen control over afferent equivalence. Overall, this framework provides a principled basis for future research, and longitudinal work should determine whether the restoration of neurovascular alignment parallels motor recovery. Accounting for these temporal shifts clarifies the interaction between neural recovery and vascular health in the post-stroke brain. Prospective, larger-scale multimodal cohorts will be necessary to validate these findings and determine whether neurovascular alignment metrics possess prognostic or therapeutic utility.

## 5 Conclusion

This study demonstrates that chronic stroke alters the temporal alignment between low-frequency EEG oscillations and task-evoked BOLD responses during finger stimulation. While raw cross-correlation analyses initially yielded negative peak lags in a subset of stroke participants, physiology-constrained lag estimation and event-locked analyses revealed that these negative values reflect oscillatory phase ambiguity and delayed hemodynamics rather than BOLD preceding neural activity. Event-locked stimulation responses showed systematically prolonged BOLD peak latency in stroke, with larger delays observed in more impaired participants. Lag estimates also showed greater dispersion in stroke, consistent with reduced temporal stability of cross-modal alignment beyond uniform delay. Canonical HRF modeling underestimated this delay, particularly in more impaired patients, whereas derivative-augmented models reduced peak-timing error and improved cross-validated generalization. These findings indicate that stroke modifies the shape and timing of the hemodynamic response function, and that failure to model this variability can produce artifactual lag interpretations in oscillatory analyses. Importantly, physiology-constrained cross-modal lag estimates restored positive EEG → BOLD delays in all participants, consistent with preserved neurovascular directionality but prolonged vascular response dynamics. Reduced ipsilesional coupling strength and delayed alignment were most pronounced in participants with lower Fugl–Meyer scores, suggesting that impaired motor recovery is associated with slowed or desynchronized neurovascular integration rather than inversion of mechanistic hierarchy. Taken together, these results support a model in which chronic stroke disrupts the timing fidelity of neurovascular coupling without reversing causal direction. The data should be interpreted as pilot evidence demonstrating delayed and spatially asymmetric coupling dynamics, rather than proof of causal inversion. Larger longitudinal studies will be required to determine the generalizability and behavioral prognostic value of these temporal alterations.

## CRediT authorship contribution statement

**Parikshat Sirpal:** Writing – original draft, Validation, Software, Methodology, Investigation, Formal analysis, Data curation. **Nishaal Parmar:** Writing – original draft, Visualization, Methodology, Data curation. **Beni Mulyana:** Methodology, Data curation. **Hazem H. Refai:** Writing – review & editing, Validation, Supervision. **Yuan Yang:** Writing – review & editing, Supervision, Resources, Project administration, Investigation, Funding acquisition, Conceptualization.

## Funding

This manuscript is the result of funding in whole or in part by the Oklahoma Center for Advancement of Science & Technology (OCAST) and National Institutes of Health (NIH R01HD109157). It is subject to the NIH Public Access Policy. Through acceptance of this federal funding, NIH has been given a right to make this manuscript publicly available in PubMed Central upon the Official Date of Publication, as defined by NIH. YY’s time on this work was also supported by his American Heart Association Award (932980) and National Science Foundation (NSF 2401215).

## Declaration of Competing Interest

The authors declare that they have no known competing financial interests or personal relationships that could have appeared to influence the work reported in this paper.

## Data Availability

Data will be made available on request.
