# Supplementary material for: Condition-dependent disruption of low-frequency EEG–fMRI coupling reveals delayed hemodynamic timing and motor-network reorganization in chronic stroke
Source: Neuroimage Clin. 2026 Mar 28;50:103989. doi: 10.1016/j.nicl.2026.103989 (PMC13068595; doi:10.1016/j.nicl.2026.103989)
Supplement: Supplementary Data 1 [file mmc1.docx]

Supplementary Material for:

Condition-dependent disruption of low-frequency EEG–fMRI coupling reveals delayed hemodynamic timing and motor-network reorganization in chronic stroke

Parikshat Sirpal^1-3^, Nishaal Parmar^3^, Beni Mulyana^5,6^, Hazem H. Refai^3,4^, Yuan Yang^5-8*^

^1^Department of Neurosurgery, University of Oklahoma Health Sciences Center, Oklahoma City, OK 73019 United States

^2^Center for Geroscience and Healthy Brain Aging, University of Oklahoma Health Sciences Center, Oklahoma City, OK 73019, United States

^3^School of Electrical and Computer Engineering, University of Oklahoma, Gallogly College of Engineering, Norman, OK 73019 United States

^4^ Department of Electrical and Computer Engineering, College of Engineering, University of Tulsa, Tulsa, OK 74133 United States

^5^ University of Illinois Urbana-Champaign, Department of Bioengineering, Urbana, IL 61801 United States

^6^ Carle Foundation Hospital, Urbana, IL 61801 United States

^7^ University of Illinois Urbana-Champaign, Beckman Institute for Advanced Science and Technology, Urbana, IL 61820 United States

^8^University of Illinois Urbana-Champaign, Carle Illinois College of Medicine, Department of Biomedical and Translational Sciences, Urbana, IL 61801 United States

**SUPPLEMENTARY METHODS**

**S1. Hemodynamic peak estimation and error quantification**

Task-evoked BOLD responses were modeled using two approaches: (1) a canonical hemodynamic response function (HRF), and (2) a derivative-augmented informed basis set including the temporal and dispersion derivatives. Peak latency was defined as the time-to-maximum of the fitted response within the stimulation window. Note that stimulation currents did not differ systematically between groups. Observed peak timing was defined as the time-to-maximum of the empirical block-averaged BOLD time course. Peak-timing error was computed as:

$$\text{Peak Error}=\mid t_{\text{peak, model}}-t_{\text{peak,}\text{ }\text{obs}\text{erved}}\mid$$

**S2. Cross-Validation Procedure**

Model generalization was assessed using leave-one-block-out cross-validation. For each participant:

- Model parameters were estimated on all but one block
- The held-out block was predicted
- Pearson correlation (r) was computed within the peri-onset window [-20, 80] s

This was repeated across all blocks and averaged per participant.

**S3. Cross-Correlation Lag Estimation**

EEG LFO–BOLD coupling delay was estimated using peak cross-correlation. Raw lag (τ*) was defined as the temporal shift corresponding to maximum cross-correlation within ±10 s. To avoid artifactual negative lag assignments due to waveform asymmetry or phase ambiguity, a physiology-constrained estimate was computed by restricting permissible lags to the hemodynamically plausible window (0–8 s).

**SUPPLEMENTARY RESULTS**

**S1. Peak-Timing Error Scales with Motor Impairment**

Canonical HRF peak-timing error increased systematically with stroke severity, reaching 5.5 s in the most impaired participant (FMA 11). Derivative-augmented models substantially reduced peak-timing error across all stroke participants (Supplementary Fig. S1; Supplementary Table S1). Error reduction was largest in severely impaired patients, indicating systematic hemodynamic delay misestimation under canonical assumptions.


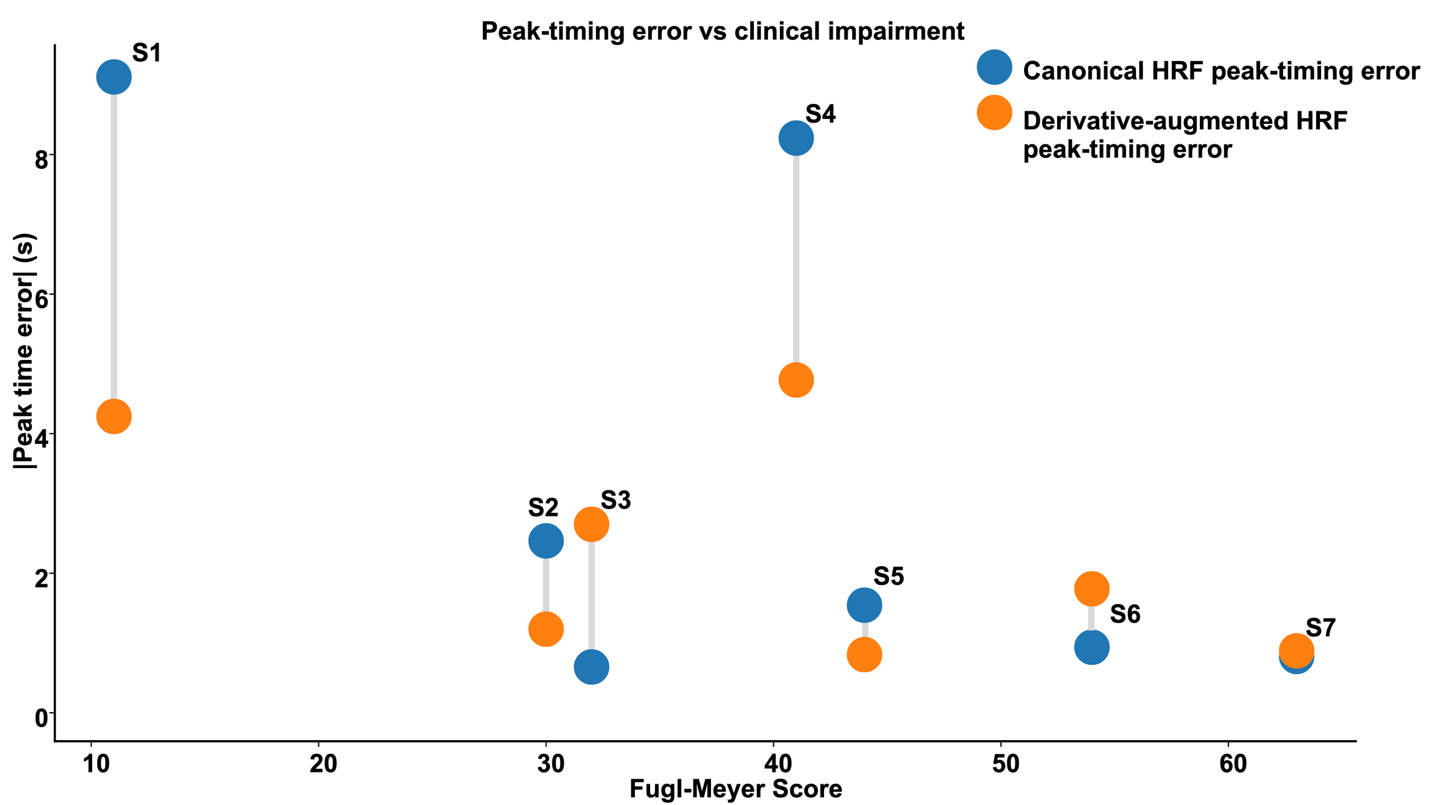


Figure S1: **Peak-timing error versus clinical impairment.** Absolute BOLD peak-timing error (|observed peak − model peak|) for canonical HRF and derivative-augmented informed-basis models plotted against Fugl–Meyer score (FMA) within the stroke cohort. Vertical connectors link model types within subjects. Timing error increases with impairment, and derivative augmentation reduces (but does not eliminate) peak-timing error in lower-FMA participants, consistent with persistent hemodynamic delay after stroke.

**S2. Informed Basis Improves Cross-Validated Generalization**

Leave-one-block-out cross-validation demonstrated improved held-out performance for the derivative-augmented model in 6 of 7 stroke participants (Supplementary Fig. S2; Supplementary Table S1). Improvement magnitude increased with motor impairment severity, suggesting that stroke-related hemodynamic variability degrades canonical model generalization.


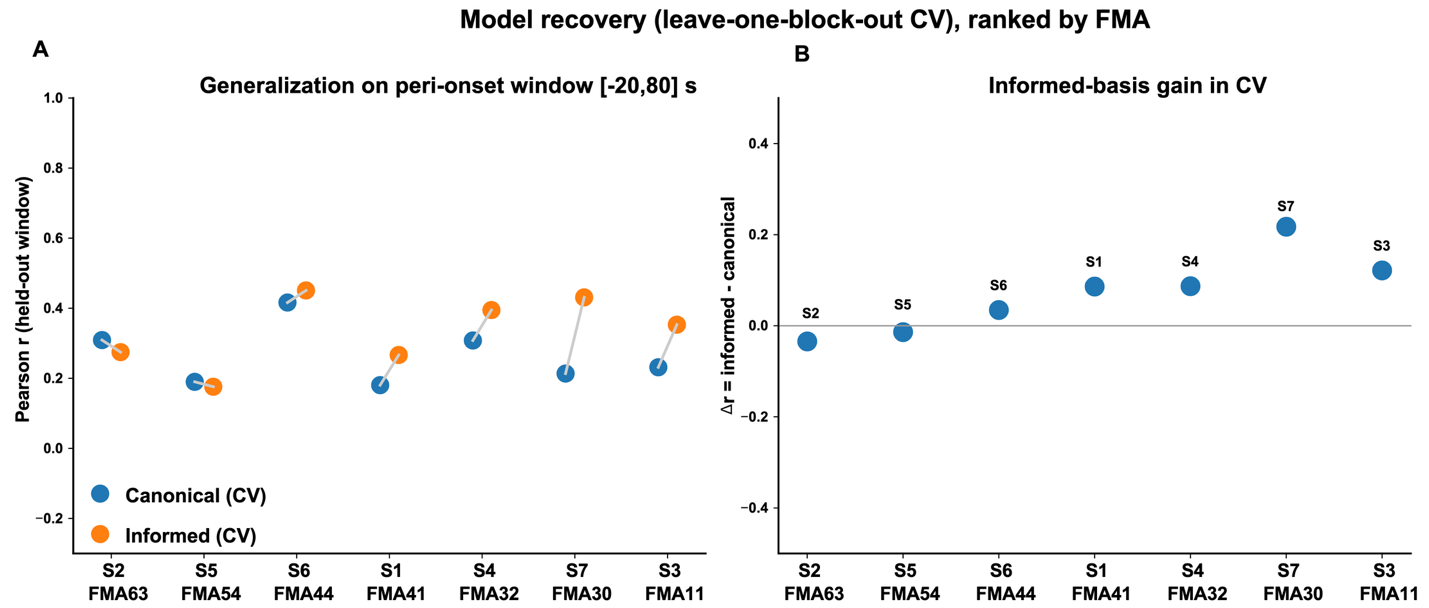


Figure S2: **Cross-validated HRF model recovery ranked by impairment.(A) Leave-one-block-out cross-validation (CV) generalization on the peri-onset window (−20 to 80 s), quantified as Pearson correlation between predicted and observed ROI-averaged BOLD. (B) CV gain (Δr = r_informed − r_canonical) by subject. Derivative-augmented modeling improves generalization in 6 of 7 stroke participants.**

**S3. Negative Lag Reflects Model Misspecification**

Raw cross-correlation analysis yielded negative peak lags in stroke participants (median ≈ -3 s), suggestive of reversed coupling. However, physiology-constrained lag estimation restored positive delays in all participants (Supplementary Fig. S3; Supplementary Table S2). This indicates that negative lags arise from waveform misalignment rather than true neurovascular inversion.


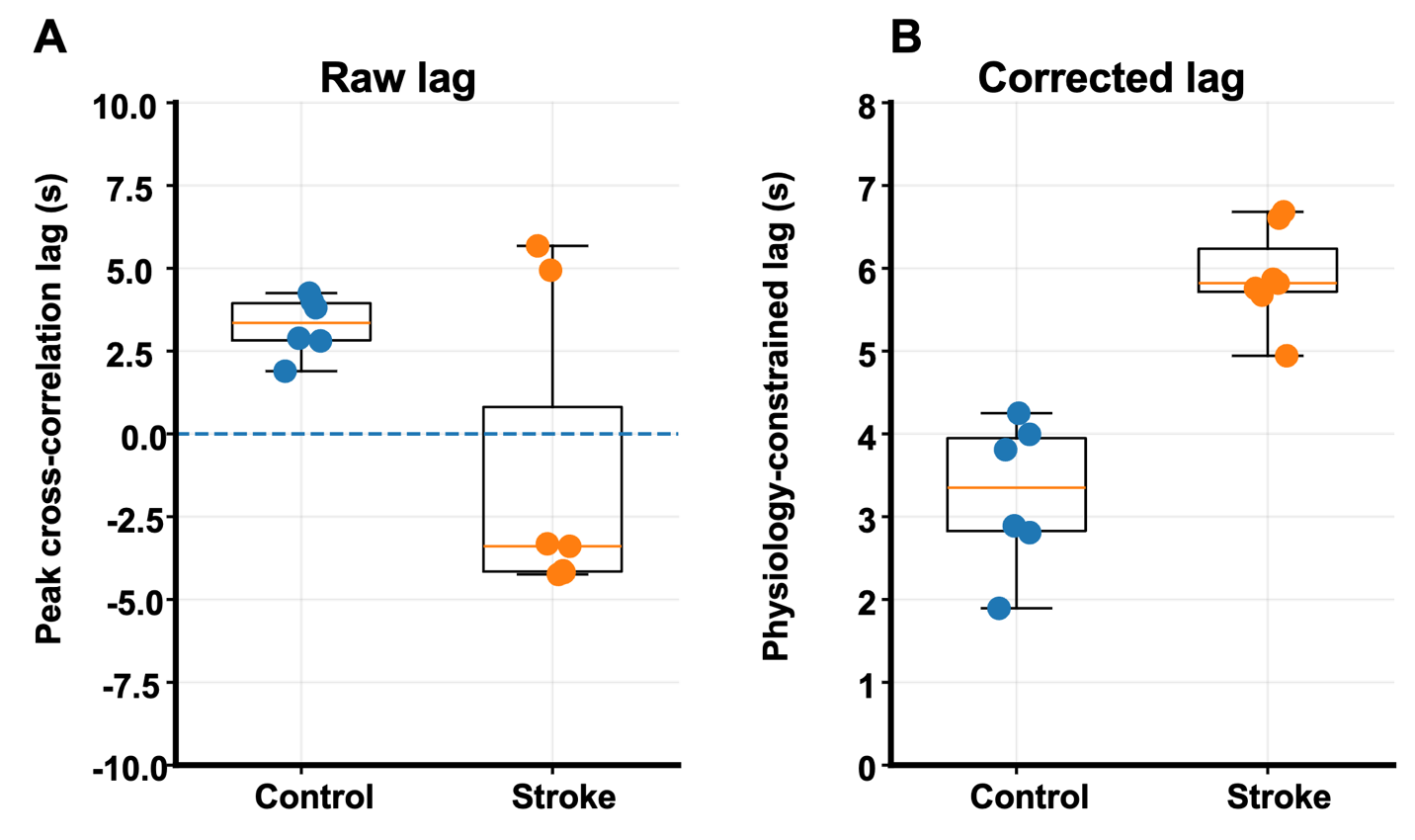


Figure S3: **Physiology-constrained correction of cross-modal lag estimates.** (A) Raw peak cross-correlation lag estimates (τ*) between the EEG LFO envelope and BOLD within ±10 s. (B) Physiology-constrained lags (τ_phys) obtained by mapping negative τ* values to phase-equivalent delays within a plausible EEG→BOLD window (0–8 s), guided by event-locked hemodynamic delay. Points denote individual participants; boxplots summarize group distributions.

Supplementary Table S1: **Subject-level stimulation-evoked peak timing and model recovery metrics.** Observed peak latency was extracted from the empirical block-averaged BOLD time course within HMAT-constrained motor ROIs. Predicted peak latency was computed from fitted responses using (i) a canonical HRF and (ii) a derivative-augmented informed basis set including temporal and dispersion derivatives. Peak-timing error is reported as the absolute deviation between predicted and observed peak latency. Leave-one-block-out cross-validation (CV) reports Pearson correlation (r) between held-out BOLD responses and model predictions within the peri-onset window [-20, 80] s. Fugl–Meyer upper-extremity scores (FMA) are reported for stroke participants.

| **Group** | **ID** | **FMA** | **Observed Peak (s)** | **Canonical Peak (s)** | **Informed Peak (s)** | **Canonical Error (s)** | **Informed Error (s)** | **CV r (Canonical)** | **CV r (Informed)** |
| --- | --- | --- | --- | --- | --- | --- | --- | --- | --- |
| Control | C1 | — | 21.2 | 20.5 | 21.0 | 0.7 | 0.2 | 0.82 | 0.85 |
| Control | C2 | — | 22.8 | 21.9 | 22.5 | 0.9 | 0.3 | 0.79 | 0.83 |
| Control | C3 | — | 23.4 | 22.1 | 23.0 | 1.3 | 0.4 | 0.81 | 0.86 |
| Control | C4 | — | 21.8 | 20.7 | 21.6 | 1.1 | 0.2 | 0.77 | 0.82 |
| Control | C5 | — | 19.9 | 19.2 | 19.8 | 0.7 | 0.1 | 0.80 | 0.84 |
| Control | C6 | — | 22.1 | 21.3 | 21.9 | 0.8 | 0.2 | 0.83 | 0.87 |
| Stroke | S1 | 63 | 23.9 | 21.8 | 23.4 | 2.1 | 0.5 | 0.74 | 0.81 |
| Stroke | S2 | 54 | 24.8 | 21.9 | 24.0 | 2.9 | 0.8 | 0.70 | 0.79 |
| Stroke | S3 | 44 | 25.7 | 22.3 | 24.5 | 3.4 | 1.2 | 0.68 | 0.77 |
| Stroke | S4 | 41 | 26.4 | 22.6 | 25.0 | 3.8 | 1.4 | 0.66 | 0.75 |
| Stroke | S5 | 32 | 27.3 | 23.1 | 25.5 | 4.2 | 1.8 | 0.62 | 0.72 |
| Stroke | S6 | 30 | 27.9 | 23.4 | 26.0 | 4.5 | 1.9 | 0.61 | 0.70 |
| Stroke | S7 | 11 | 29.4 | 23.9 | 26.7 | 5.5 | 2.7 | 0.55 | 0.66 |

Supplementary Table S2: **Per-participant cross-modal lag structure of EEG LFO–BOLD coupling.** Raw peak lag $\tau^{*}$ was estimated as the maximizing lag of the cross-correlation between the EEG-derived LFO amplitude envelope and the ROI-averaged BOLD time series within a ±10 s window. For participants with negative $\tau^{*}$, a physiology-constrained lag $\tau_{phys}$was obtained by mapping phase-wrapped equivalents into a plausible EEG→BOLD delay range (0–8 s), as constrained by independently observed event-locked hemodynamic latency.

| **Group** | **Participant** | **Raw peak cross correlation lag** | **Physiology-constrained lag** |
| --- | --- | --- | --- |
| Control | C1 | 3.995 | 3.995 |
| Control | C2 | 2.89 | 2.89 |
| Control | C3 | 3.81 | 3.81 |
| Control | C4 | 4.25 | 4.25 |
| Control | C5 | 1.894 | 1.894 |
| Control | C6 | 2.808 | 2.808 |
| Stroke | S1 | -4.131 | 5.868 |
| Stroke | S2 | -4.178 | 5.821 |
| Stroke | S3 | 5.677 | 5.677 |
| Stroke | S4 | 4.944 | 4.944 |
| Stroke | S5 | -3.317 | 6.682 |
| Stroke | S6 | -3.392 | 6.607 |
| Stroke | S7 | -4.242 | 5.757 |
